# Leprosy Mimicking Common Rheumatologic Entities: A Trial for the Clinician in the Era of Biologics

**DOI:** 10.1155/2014/429698

**Published:** 2014-11-06

**Authors:** Deepak Rath, Shrinath Bhargava, Bijit Kumar Kundu

**Affiliations:** ^1^Rheumatology Clinic, Department of Medicine, PGIMER & Dr. RML Hospital, New Delhi 110001, India; ^2^Department of Dermatology, Dr. RML Hospital, New Delhi 110001, India

## Abstract

Rheumatoid arthritis and seronegative spondyloarthritis, which make up the lion's share of cases attending a rheumatology clinic, are relatively easy to diagnose. However, when an entity of infective aetiology like leprosy known to be a great mimic of different autoimmune conditions presents with features similar to these, the possibility of it being diagnosed at the outset is very slim indeed. The ease with which the diagnosis of leprosy can be missed assumes sinister proportions as the use of disease modifying agents can have deleterious effects in these patients. In the era of increasing availability and use of biologic disease modifying agents, it is imperative not only to actively rule out the presence of leprosy but also to make it a part of the prebiologic screening of patients in whom biologics are being planned to be administered, especially in leprosy endemic areas.

## 1. Introduction

Affection of skin and nerves by leprosy is quite common and is easily diagnosed. However diagnosing musculoskeletal involvement as being due to leprosy is difficult, given its protean manifestations and the fact that it is a great mimic of many autoimmune conditions. We present two cases in which leprosy presented with features which were the same and were thus initially diagnosed and treated as rheumatoid arthritis (RA) and seronegative spondyloarthritis (SpA), respectively. Only later was the true diagnosis revealed. In areas endemic for leprosy it is imperative to keep in mind the myriad presentations of leprosy including its musculoskeletal manifestations, as consequences of misdiagnosis and hence mistreatment can be catastrophic, especially in the era of increasing use of biologic disease modifying agents. We conclude that in endemic areas leprosy should be “actively” ruled out and should be made an integral part of prebiologic screening.

## 2. Case I

Mrs. KY, a 35-year-old lady, presented to our rheumatology clinic with the chief complaints of gradual onset slowly progressive polyarthritis affecting the small and large joints of hands in “rheumatoid” distribution, along with nodules over her lower legs on the extensor aspect. The polyarthritis was associated with early morning stiffness which would decrease with activity. Review of the history revealed that she initially had arthritis of wrist followed by knees and ankles a year and half back. The affection of knees and ankles was transient. This was followed a few months later by eruption of nodules on the shins accompanied by feverish feeling. The nodules would resolve after a few days leaving behind a hyperpigmented macule. New nodules would subsequently appear and follow the same pattern. Arthritis of the small joints of the hands had developed a few months back, almost a year after the affection of the wrist. She had been diagnosed as seronegative RA and treated by a practitioner of alternative medicine, with resolution of symptoms for 3-4 months. She developed intolerance to the medications and had to stop the treatment, but the arthritis and nodules relapsed with a severity more than before. She had presented to the department of medicine of our hospital where she was evaluated initially as a case of polyarthritis with nodules. Her investigations revealed normal haemogram (haemoglobin (Hb): 11.8 gm/dL; total leucocyte count (TLC): 7000/cmm; DC-N79 L35 E6; platelet count (TPC): 280000/cmm) and erythrocyte sedimentation rate (ESR) of 29 mm/1st hour. Liver and kidney function tests were within normal limits as was the examination of the urine. Rheumatoid factor (RF), C-reactive protein (CRP), and anti-cyclic citrullinated peptide antibodies (anti-CCP) were negative by slide agglutination and nephelometry, respectively. Serum angiotensin converting enzyme (ACE) levels too were within normal limits (23 U/L, normal: 8–65 U/L). However, anti-nuclear antibodies (ANA) were found to be positive (slide agglutination). She was referred to our rheumatology clinic with the diagnosis of seronegative RA.

**Figure 1:**
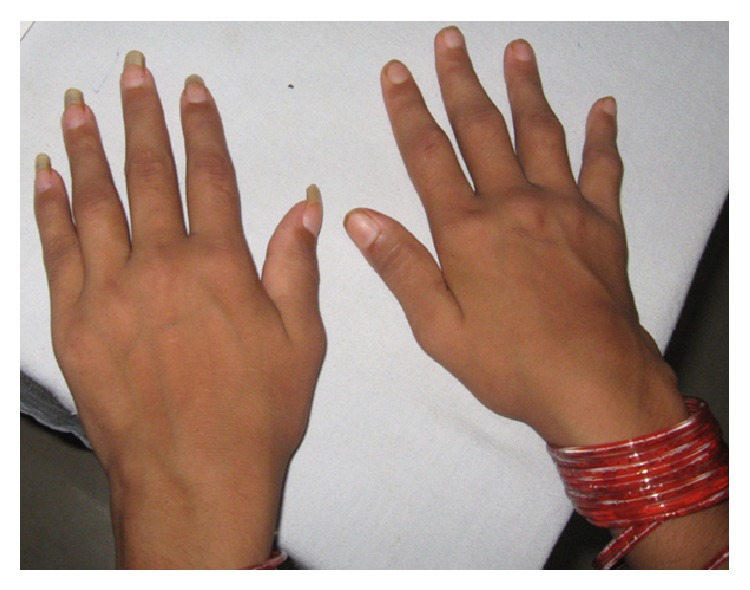
Symmetrical synovitis of wrists, ankles, and small joints of the hand.

Examination in our clinic revealed a person, normal in build and appearance, with normal vitals. Skin examination revealed few lesions resembling resolving erythema nodosum and hyperpigmented macular lesions where previous lesions had existed on the extensor surfaces of the legs. The rest of the general and systemic examination was unremarkable. Musculoskeletal examination revealed bilaterally symmetrical synovitis of wrists, ankles, and small joints of the hand (Figure 1). A provisional diagnosis of seronegative rheumatoid arthritis was kept along with differentials of tuberculosis/sarcoidosis based on the profile of patients presenting to our rheumatology clinic with similar manifestations. Montoux test was nonreactive, and CECT of the chest did not show any hilar lymphadenopathy. Dermatologist opined the nodular swelling to be erythema nodosum (EN). She was started on Hydroxychloroquine 200 mg twice daily and NSAIDs. After a few weeks, she developed increase in the number, frequency, and severity of the pains of the EN. A biopsy of the nodule was done elsewhere and reported as EN. She was advised Deflazacort 15 mg per day, which she took for 10 days, without any improvement in either her joint pains or her skin lesions. She reported back to our clinic, where we found synovitis of the wrists and small joints persisting, but an increase in number and spread of nodules to the upper arms and face. This led us to the suspicion of leprosy and the possibility of the skin lesion being erythema nodosum leprosum (ENL) and not EN. Though she did not have any skin lesion to suggest leprosy like hypopigmented and/or hypoaesthetic patches, xerosis, induration of pinnae or ear lobes, and madarosis, we found thickened and tender ulnar and radial cutaneous nerve bilaterally and common peroneal nerve and posterior tibial nerve on the left side but without any sensory loss in the areas supplied by these nerves. A deep biopsy of the nodules and slit smears examination from ear lobes and forehead were done. The biopsy of the nodule at our centre revealed hyperkeratosis, acanthosis with flattened rete ridges, perivascular and appendegeal collection of neutrophils lymphocytes, epithelioid cells, and foamy histiocytes in the mid and lower dermis and along the junction of dermis and subcutis. The infiltrate extended into the erector pili muscle and subcutis. Occasional medium sized vessels revealed evidence of vasculitis. Fite staining showed few fragmented acid-fast bacilli (AFB). Bacteriological Index (BI) of 3+ AFB was reported from the slit skin smears. She was thus diagnosed with Hansen's disease presenting as gradual onset chronic polyarthritis and ENL. She was put on multidrug therapy (MDT), along with steroids, following which she recovered.

## 3. Case II

Mr. PP, a 40-year-old gentleman, presented to the hospital with a history of gradual onset, progressive joint pains in additive pattern affecting the large joints (knees, ankles, and shoulders) asymmetrically along with inflammatory low back pain (ILBP) over the past two and half years, multiple nodular swellings of size 1-2 centimetres all over the body, and few red plaques over his back and left knee, associated with low grade fever for the past one and half years. Response to NSAIDs was not satisfactory. There were no other clinical seronegative spondyloarthritis (SpA) features.

**Figure 2:**
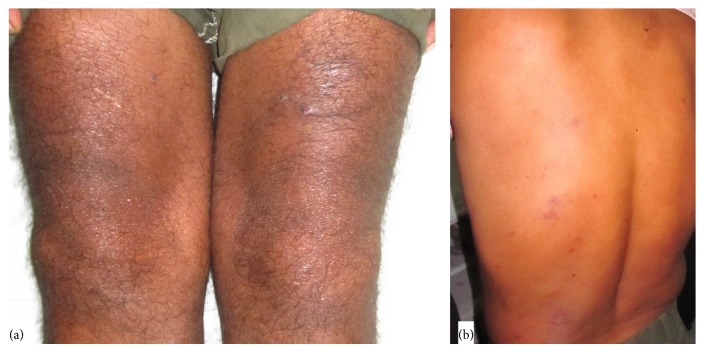
(a) Shiny, plaque lesion over left knee diagnosed as steroid modified psoriasis. (b) Erythematous, plaque like lesions over the back thought to be plaque psoriasis.

The swellings were gradual in onset, discreet, slowly progressive, and occurring over multiple areas of the body without any specific pattern of affection or sparing and without any history of redness, pain, or discharge. Neither he nor anyone in his family ever had tuberculosis. His investigations done outside revealed high ESR of 79 and elevated CRP of more than 32 mg/dL. The erythematous plaques had been diagnosed and treated elsewhere as psoriasis with local steroids (Figure 2). Based on the lower limb large joint arthritis, ILBP, psoriasis, radiological sacroilitis, and elevated CRP, he was diagnosed with SpA and psoriatic arthritis, with steroid modified psoriasis, and prescribed NSAIDs and sulfasalazine (SSZ). As he had not shown any improvement in symptoms, he was referred to our clinic with suggestion to consider biologics (antitumor necrosis factor alpha (anti-TNF-*α*)).

**Figure 3:**
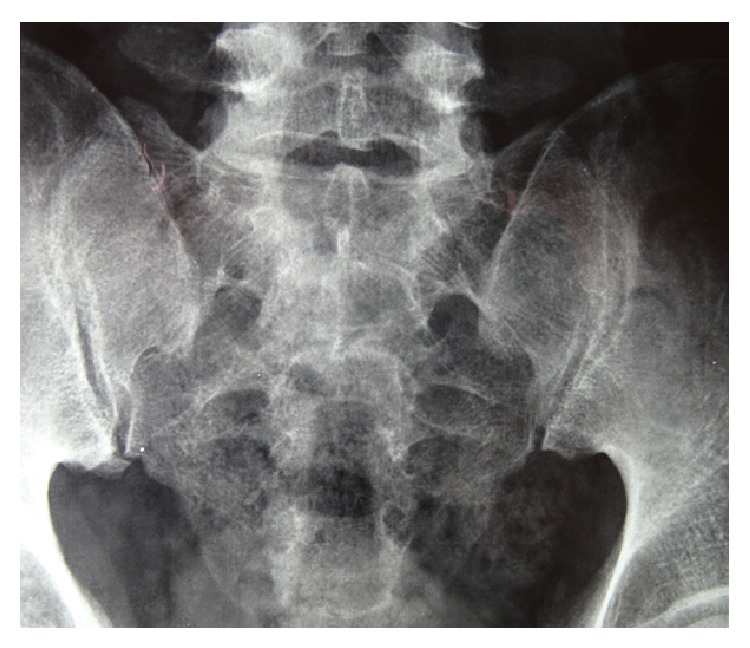
Sacroilitis.

Based on the history and clinical examination, provisional diagnosis of SpA and psoriatic arthritis was kept, and his previous investigations were reviewed. Rheumatoid factor (RF), anti-CCP, ANA, human leucocyte antigen B27 (HLA B27), human immunodeficiency virus (HIV) I and II, hepatitis B surface antigen (HBsAg), anti-hepatitis C viral antibody, and* Brucella* antibodies (IgG and IgM) were all negative. Serum ACE levels were within normal limits (61.1 mg/dL, normal: 8–65 mg/dL). FNAC of axillary lymph node showed features of chronic inflammation. X-ray of pelvis showed bilateral sacroilitis (Figure 3).

General examination revealed generalized lymphadenopathy, with firm, nontender, discreet lymph nodes palpable in inguinal and cervical regions bilaterally and in the right axillary region with the largest being around 2 cm. Examination of the skin showed multiple nodular lesions over the legs, trunk, and back. Erythematous plaques were also seen over the back, knees, and shins. Musculoskeletal examination revealed asymmetrical synovitis of the wrists, knees, and ankles along with tenosynovitis of the feet. The character of pain suggested a neuritic origin and prompted his neurological examination. He was found to have thickened but nontender ulnar and peroneal nerves with evidence of sensory deficit in the distribution of the nerves. However other leprosy features like induration of earlobes and madarosis were conspicuous by their absence.

**Figure 4:**
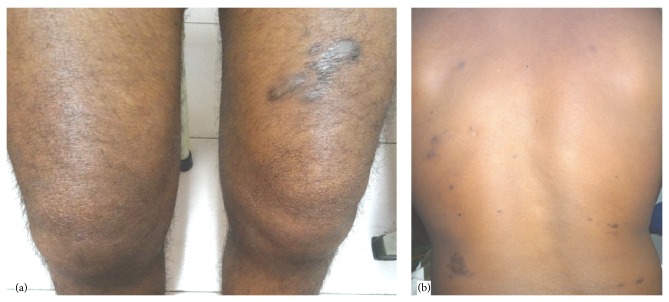
(a) Lesion shown in Figure 2(a) after 1-year treatment of leprosy. (b) Lesion shown in Figure 2(b) after 1-year treatment of leprosy.

Dermatology review was undertaken and a possibility of leprosy (BL) with ENL was kept. The BI of the slit skin smear of the patient was reported as 3+. Biopsy of nodules revealed noncaseating granulomas with occasional epithelioid cells arranged in patches. Multiple acid fast bacilli were visualized with Fite stain. He was put on MDT and steroids and showed marked improvement in his symptoms, both articular and dermatological (Figure 4).

## 4. Discussion

Leprosy or Hansen's disease, caused by* Mycobacterium leprae* (*M. Leprae*), continues to be a public health problem in endemic regions including India, though detection of new cases has decreased from 260063 in 2004 to 127295 in 2011 [1].

Though the classical manifestations affecting the skin and nerves are well known in general, what has come to the attention in recent times is its “atypical” presentation with affection of the musculoskeletal system in its various forms as the primary presentation, with dermatological and neural manifestations occurring later and sometimes not at all. The “atypical” musculoskeletal affection does not include the classical neuropathic joints of Charcot's taught to medical students, now rarely seen.

Musculoskeletal affection by leprosy is varied and can mimic RA, SpA, and even vasculitis [2]. The likelihood of its misdiagnosis and thus mistreatment, leading to potentially disastrous consequences, is high in this scenario. The risk is increased manyfold with the increasing availability and use of biologic disease modifying agents especially TNF*α* inhibitors. Anti-TNF agents cause death of cells expressing TNF and disrupting granulomas which lead to reactivation of granulomatous disease. Though attention has been focused more on TB, reactivation of leprosy has also been reported [3].

Leprosy can be of five types, namely, tuberculoid (TT), borderline tuberculoid (BT), borderline (BB), borderline lepromatous (BL), and lepromatous (LL), which correspond to decreasing levels of immunity from TT to LL. Arthritis occurs in more than half of the cases and is seen in all types but most commonly in BL, followed by TT [4]. The Indian classification has an additional type without skin lesions, called the pure neuritic type, which too can present with arthritis [5].

A characteristic of leprosy is lepra reaction, which can be of three types. Type I can be downgraded with worsening towards lepromatous features, usually before therapy or the reversal reaction occurring due to the improvement of cell mediated immunity shifting the type from borderline to tuberculoid, usually due to treatment. It presents as fever, inflamed skin, tender peripheral nerves, and sometimes arthritis [6]. Type II reaction, also called ENL, is seen in the BL and LL types and is an immune complex disease appearing histologically as a polymorphonuclear vasculitis. It presents as painful red nodules which may ulcerate, along with fever, malaise, and joint and neuritic pains and eye and other organ involvement including lymphadenopathy and orchitis. Type III lepra reaction or the Lucio phenomenon is a cutaneous vasculitis seen in lepromatous leprosy affecting people who are incompliant with treatment. It presents as odd-shaped red patches and ulcers on extremities and is associated with liver and kidney disease in addition to fever and arthritis [7, 8].

Leprosy as well as lepra reactions can be accompanied by arthritis. Arthritis is well known to be classically associated with the lepra reactions, especially Type II, with the incidence being more than 57% [4]. The onset in these cases is usually acute, corresponding to change in immune status of the patient, usually with treatment, and affecting the small joints of the hand and feet symmetrically in the so-called “rheumatoid distribution,” subsequently resolving over a few weeks. Chronic symmetrical small joint arthritis resembling RA without being associated with lepra reaction is also known to occur [9].

Patients of leprosy have a number of autoantibodies which add to the confusion. These include CRP, antistreptolysin-O (ASO), RF, ANA, anti-neutrophil cytoplasmic antibodies (ANCA) [4], and even anti-phospholipid antibodies, double stranded deoxyribonucleic acid (ds-DNA), and anti-CCP. The incidence of anti-CCP is, however, reported to be lower [10]. Needless to say these autoantibodies are found more towards the lepromatous pole of the disease.

We have presented two cases with features similar to RA and SpA, respectively. Our first case with gradual onset symmetrical small joint polyarthritis was initially diagnosed as seronegative RA. The nodules appeared later and were initially localized over the shins, prompting a diagnosis of EN. The ANA too was positive at our centre. All these findings “gelled” with each other in the initial diagnosis of seronegative RA. Leprosy was only doubted when the nodules started appearing over the face, a site not affected by erythema nodosum.

In our second case, the pattern of lower limb large joint asymmetrical arthritis, ILBP, erythematous plaques suggestive of psoriasis, elevated CRP, and radiological sacroilitis suggested the initial diagnosis of SpA. Leprosy was diagnosed on basis of neuritic pain, thickened nerves, and histopathology studies.

RA and SpA make up the bulk of cases attending any rheumatology clinic and thus the probability of missing a case of leprosy presenting with similar features is extremely high. The arthritis of leprosy may resemble the pattern of joint affection in RA or SpA [2] as has been shown in our cases. It is also known to resemble vasculitis and sarcoidosis. Further confounding the issue is the presence of autoantibodies relevant to different arthritides in leprosy also.

Few points to distinguish the aetiology as leprosy are absence of rheumatoid nodules and other extra articular features and the low probability of anti-CCP positivity. It is also important to distinguish EN and ENL. Though they can be very similar clinically, ENL is usually more in number and occurs in areas like arms, trunk, and face, while EN is fewer and occurs mostly on the shins, and hence presence of nodules in areas apart from the legs should prompt suspicion of it being ENL instead of EN. The lack of response or worsening symptoms on treatment with disease modifying agents should prompt a review of diagnosis. Nothing however can substitute a stringent awareness of the protean manifestations of leprosy and a thorough clinical examination intended specifically to rule out leprosy especially in endemic areas.

We conclude that leprosy should be considered as a differential diagnosis while evaluating any case of arthritis especially in leprosy endemic areas and should be actively ruled out clinically and if necessary by investigations. This assumes utmost importance in view of increasing use of TNF-*α* inhibitors and hence should be made an integral part of “biologic screening” prior to institution of biologic therapy.
